# Factors lead to return to sports and recreational activity after total knee replacement: A retrospective study

**DOI:** 10.1051/sicotj/2020009

**Published:** 2020-05-07

**Authors:** Jeremy Plassard, Jean Baptiste Masson, Matthieu Malatray, John Swan, Francesco Luceri, Julien Roger, Cécile Batailler, Elvire Servien, Sébastien Lustig

**Affiliations:** 1 FIFA Medical Center of Excellence, Department of Orthopaedic Surgery and Sports Medicine, Croix Rousse Hospital, Civil Hospices of Lyon 69004 Lyon France; 2 LBMC UMR T 9406, Laboratory of Chock Mechanics and Biomechanics, Claude Bernard Lyon 1 University 69100 Villeurbanne France; 3 LIBM – EA 7424, Interuniversity Laboratory of Biology of Mobility, Claude Bernard Lyon 1 University 69100 Villeurbanne France; 4 Department of Orthopedic and Traumatology, Dijon University Hospital 21000 Dijon France; 5 Clinica Ortopedica, ASST Centro Specialistico Ortopedico Traumatologico Gaetano Pini-CTO Piazza Cardinal Ferrari 1 20122 Milan Italy; 6 Laboratory of Applied Biomechanics, Department of Biomedical Sciences for Health, Università degli Studi di Milano Via Mangiagalli 31 20133 Milan Italy

**Keywords:** Total knee replacement, Return to sport, Recreational activities, Patient satisfaction

## Abstract

*Introduction*: The number of total knee replacements performed (TKR) is increasing and so are patient expectations and functional demands. The mean age at which orthopedic surgeons may indicate TKR is decreasing, and therefore return to sport (RTS) after TKR is often an important expectation for patients. The aim of this study was to analyze the mid-term RTS, recreational activities, satisfaction level, and forgotten joint level after TKR. *Methods*: Between January 2015 and December 2016, 536 TKR (same implant design, same technique) were performed in our center. The mean age at survey was 69 years with a mean follow-up of 43 months. All patients who did not have a follow-up in the last 6 months were called. Finally, 443 TKR were analyzed. RTS was assessed using the University of California Los Angeles Scale (UCLA), forgotten joint score (FJS), and Satisfaction Score. *Results*: In this study, 85% of patients had RTS after TKR with a mean UCLA score increasing from 4.48 to 5.92 and a high satisfaction rate. Satisfaction with activity level was 93% (satisfied and very satisfied patients). The RTS is more important for people with a higher preoperative UCLA score and a lower American Society of Anesthesiologist score (ASA). Each point increase in ASA score is associated with reduced probability to RTS by 52%. *Discussion*: RTS and recreational activity were likely after TKR with a high satisfaction score. Preoperative condition and activity are the two most significant predictive factors for RTS. *Level of evidence*: Retrospective case series, level IV.

## Introduction

According to demographic projections, the number of patients suffering from osteoarthritis (OA) will increase dramatically in the future, and therefore the number of total knee replacement (TKR) too. This increase is multifactorial: improved life expectancy, increasing obesity, and younger patients with knee OA secondary to trauma or other reasons. The younger population often expects to maintain an active lifestyle without pain, or stiffness.

OA restricted the ability to carry out daily activities, work, and perform sport activities. Pain and decreased range of motion lead to physical deconditioning with reduced endurance, less aerobic capacity, and a higher risk of being overweight and developing cardiovascular diseases. Ravi et al. [1] reported how the management of knee OA with arthroplasty may have a role in cardiovascular prevention. Furthermore, TKR is a cost-effective surgical procedure, especially in young and active patients [2].

TKR provides functional improvement in patients with advanced knee OA [3, 4]. Although evidence suggests a full return to active life after TKR is possible [5, 6], many orthopedic surgeons still prefer to advise limited sporting activities postoperatively.

In fact, pain reduction is often insufficient with young highly demanding patients with OA wanting to return to the same pre-arthritic level of sport. These patients do not only aim for pain relief after TKR. With innovative surgical techniques and more anatomical implants, TKR is now indicated in younger patients with higher sports expectations after TKR [7]. Nowadays it is becoming more important to be able to predict the level of postoperative sport activity for each specific patient. Bullens et al. [4] and Nobel et al. [8] compared subjective and objective outcomes after TKR, and notably, satisfaction depends on pre-operative expectations, which are correlated with objective results.

The aim of this study was to analyze the mid-term return to sport (RTS) and recreational activities, satisfaction level, and forgotten joint level, and to find preoperative factors associated to better functional outcomes after TKR, then, to know whether these factors could allow a better preoperative information to patients about RTS and expected activities after TKR.

## Material and methods

### Study design

There were 536 patients enrolled in this retrospective study who underwent TKR between January 2015 and December 2016. Surgery was performed using the same primary high-flexion posterior-stabilized cemented implant (Anatomic^©^, Amplitude, France).

TKR was performed without tourniquet using the same surgical technique by four different senior surgeons. A medial parapatellar trans-quadricipital approach was performed for varus knees and the Keblish lateral approach was performed for valgus knees. The patella was resurfaced only when severe patellar osteoarthritis was present. An immediate full-weight-bearing rehabilitation protocol was used with thromboembolic prophylaxis postoperatively for 30 days in all patients.

**Figure 1:**
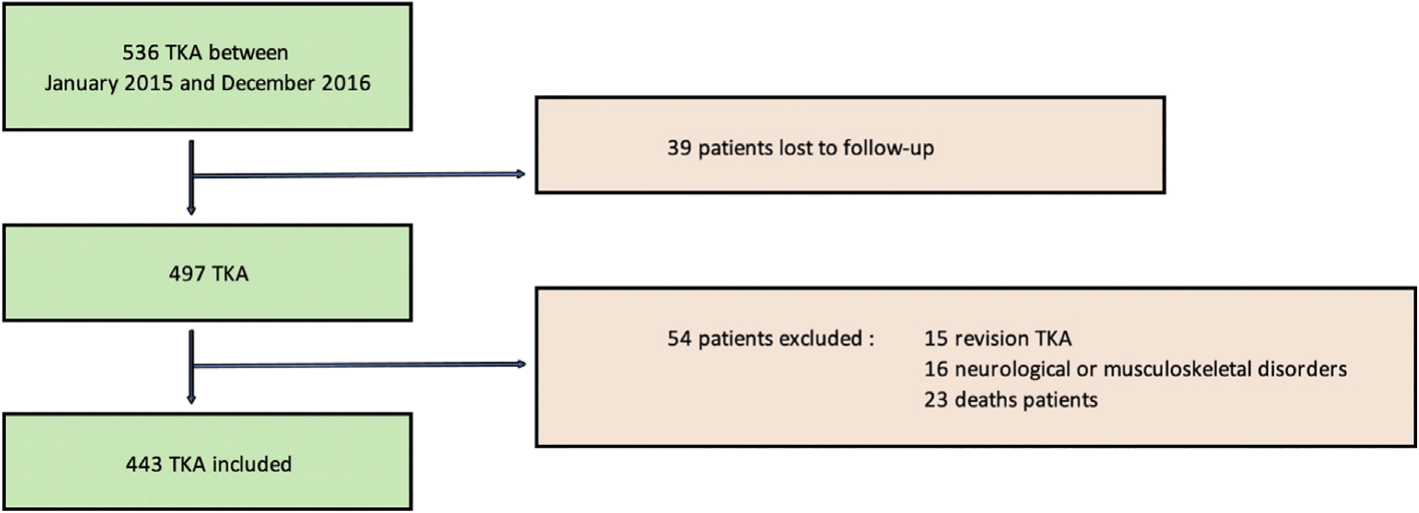
Flowchart of the study (RTKR: revision TKR).

The inclusion criteria were primary TKR for symptomatic knee osteoarthritis. A total of 536 TKR were performed between January 2015 and December 2016. The exclusion criteria were: revision TKR, inflammatory osteoarthritis, neuromuscular conditions (Parkinson’s disease, Alzheimer’s disease, stroke), or conditions that may interfere with the standard postoperative rehabilitation protocol. We excluded 15 revision TKR, 16 patients with neurological or musculoskeletal disorders and 23 deaths at the time of final follow-up. All patients without clinical follow-up in the last 6 months were evaluated using a functional questionnaire. Finally 39 patients (7%) were lost to follow-up (non-responders or contact details where changed). Four hundred and forty three TKR were included in the study. Patient flow-chart is presented in Figure 1 and demographic characteristics are summarized in Table 1. The mean follow-up in this study was 43 months [23–49 months].

**Table 1:** Sample characteristics and outcomes.

Age (years)	69 (41–90)
Age < 65 yo	146 (41–65)
Age > 65 yo	297 (66–90)
Gender	
Male	162 (36.6%)
Female	281 (63.4%)
BMI	29.3 (19–46)
Knee surgery before	
Medial meniscectomy	58 (13%)
Articular washing	38 (8.5%)
HTO	32 (7%)
ACL ligamentoplasty	27 (6%)
Lateral meniscectomy	24 (5.5%)
Tibial plate fracture	10 (2.2%)
Temporal fracture	4 (1%)
DFO	1 (0.2%)
Varus deformity	311 (70%)
Valgus deformity	87 (20%)
Normoaxis	45 (10%)
IKS objective	47
UCLA < 4	119 (27%)
UCLA 4–6	289 (65%)
UCLA > 6	35 (8%)

Patients completed the questionnaire at 2 months, 1-year follow-up, and every 2 years postoperatively. Before surgery activity level was assessed using the UCLA score [3, 9] and sport type by direct question. All patients who did not have face-to-face consultation in 2019 were called. We analyzed the postoperative UCLA score: activity level: low (≤3), moderate (4–6), and high (≥7) [10]. RTS after surgery (in months), type of sport most frequently performed after surgery, forgotten joint score (FJS) [11] and a satisfaction score (very satisfied, satisfied, disappointed, dissatisfied) based on the new IKS score [12] were also collected in the study.

### Statistical analysis

Patient demographics were described using means and standard deviations or medians and ranges for continuous variables and percentage counts for categorical variables. The study sample was divided into four sub-groups according to sex and age: younger group (age ≤65 yo) and older group (age > 65 yo). Preoperative and postoperative sport level, RTS, FJS, and the level of satisfaction were compared between groups. A multivariate linear regression analysis was performed to assess possible relationships between return to sport and the included variables: age at time of surgery, ASA score, sex, body mass index (BMI), preoperative UCLA group (low, moderate, or high), type of deformity (neutral, varus, or valgus alignment groups), degree of deformity, and patellar resurfacing. We used a generalized binomial linear logistic model produced under R Commander for multivariate analysis.

## Results

### Return to sport

A total of 376 patients (85%) returned to sport after TKR, 240 women (85%) and 136 men (84%). This represents 88% patients in the ≤65 yo group (129 patients) and 83% in the >65 yo group (247 patients). The mean delay to RTS was 5 months [1–36 months], with 33% of the patients returning to sport or activities within 3 months postoperatively and 81% of the patients within 6 months postoperatively.

### Predictive factors to RTS

Multivariate analysis shows a significant difference in return to sport according to preoperative UCLA scores (*p* < 0.001). Patients with a higher preoperative UCLA score had a higher RTS rate. A higher ASA score was a negative predictive factor for RTS (*p* < 0.005), with each increase in ASA of one point being associated with a reduction of RTS probability by 52%. There were no significant differences in age, sex, BMI (*p* = 0.054), severity of preoperative knee deformity, previous knee surgery history, and patellar resurfacing.

### Level of activity: UCLA

**Figure 2:**
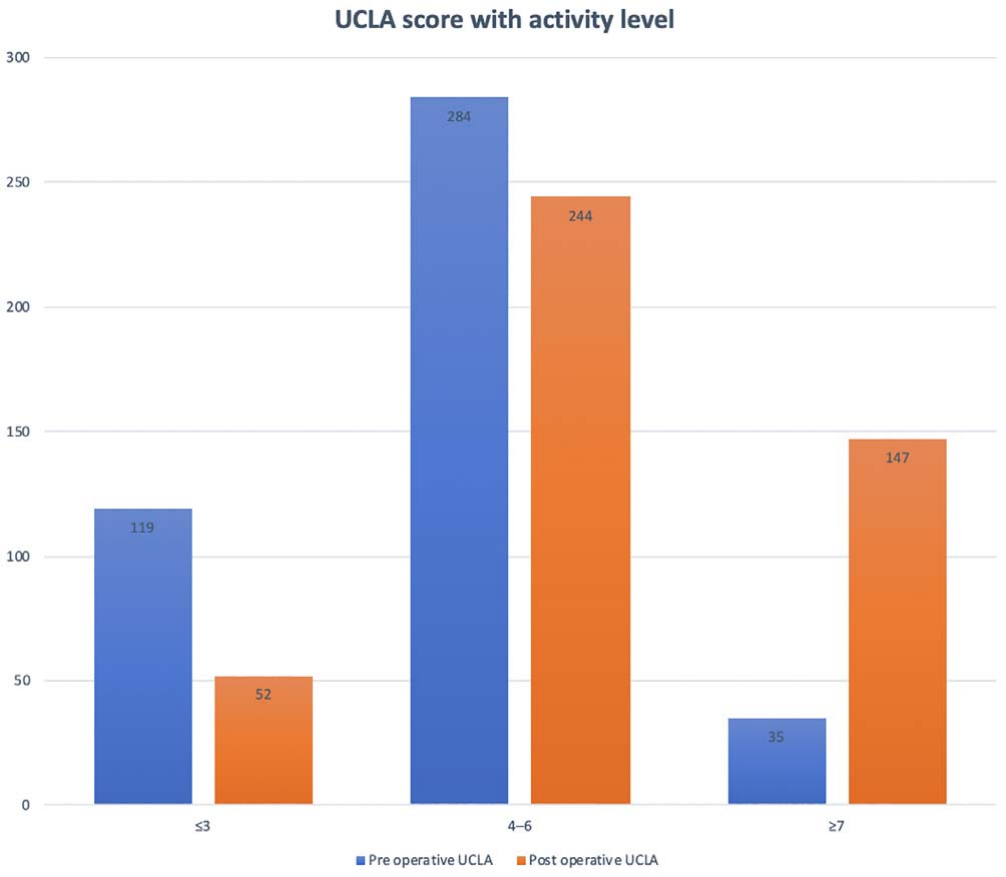
Pre- and post-operative UCLA score with activity level.

**Figure 3:**
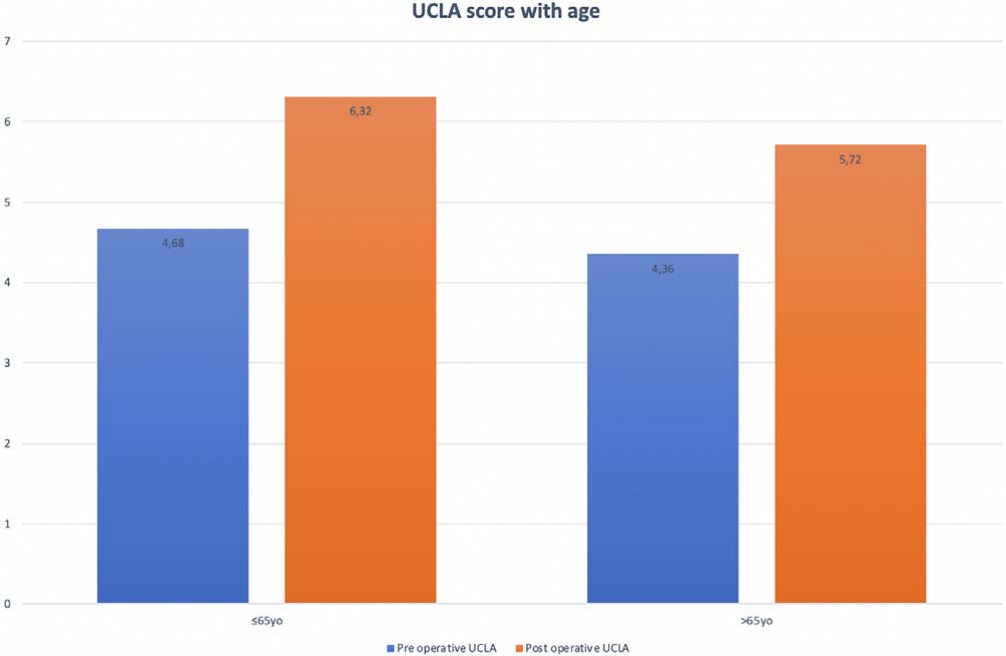
Pre- and post-operative UCLA score with age.

All patients were divided into three groups for activity level: low activity group (UCLA score ≤ 3), moderate group (UCLA score 4–6), and high active group (UCLA scale ≥ 7) (Figure 2); and in two groups for sex and age: ≤65 years and >65 years (Figure 3). The mean preoperative UCLA score was 4.45 and the mean postoperative UCLA score was 5.92, the mean UCLA activity score increased by 1.47 point. Compared to preoperative UCLA scores, 357 patients (80%) had a significant postoperative improvement, 64 patients (14%) achieved the same score, and 22 patients (5%) had a decreased UCLA score (1–3 UCLA points). After TKR, 82% of patients declared being RTS or recreational activities, with only 67 patients declaring restriction in their daily activities due to their TKR.

### Satisfaction and forgotten joint score (FJS)

At the last follow-up, the satisfaction rate after TKR was important. Four hundred and twelve patients reported that they were satisfied or very satisfied (93%), 17 were moderately satisfied (4%), and 15 were unsatisfied (3%). Unsatisfied patients had a lower FJS level, down to 25/100 and only 12 patients returned to sport. 344 patients (77%) had a FJS higher than 75/100 and no limitations in physical activity and their satisfaction rate was up to 38/40. There were no significative differences in postoperative satisfaction between gender and age.

### Sport disciplines

The most frequently performed activities were walking, hiking, gardening, swimming, yoga, cycling, and playing golf (Table 2). Three hundred and seventy six patients were involved in sports activities, 326 patients (86.9%) reported achieving a higher level. 39 patients reported being at the same level (10.3%) and 11 patients (3%) at a lower level compared to preoperative levels. Low-impact activities such as walking, hiking, cycling, or swimming were performed more after surgery and we found a decrease in high-impact activities such as tennis, jogging, or skiing. Patients with a lower level of sporting activity cited reasons such as precautionary avoidance to preserve their TKR (nine patients), knee pain (seven patients), general health condition, two patients with the sensation of knee instability, and two patients with periprosthetic infection.

**Table 2:** Variation in types of sport practiced.

	Pre operative	Post operative
Walking	208 (46.9%)	263 (59.3%)
Cycling	106 (23.9%)	120 (27.0%)
Swimming	47 (10.6%)	56 (12.6%)
Gardening	50 (11.3%)	55 (12.4%)
Hiking	48 (10.8%)	54 (12.2%)
Fitness/yoga	26 (5.8%)	32 (7.2%)
Skiing	28 (6.3%)	23 (5.2%)
Golf	11 (2.4%)	9 (2.0%)
Petanque	6 (1.3%)	6 (1.3%)
Fishing/hunting	4 (0.9%)	5 (1.2%)
Tennis	5 (1.2%)	3 (0.7%)
Dancing	0	2 (0.5%)
Running	4 (0.9%)	1 (0.3%)
Climbing	1 (0.3%)	1 (0.3%)

### Range of motion evolution

We analyzed for an association between range of motion (ROM) and patient satisfaction. In unsatisfied, low-satisfied, and satisfied groups, the range of motion decreased after TKR: from 117° to 109° for unsatisfied patients, from 122° to 114° for low-satisfied patients, and from 125° to 122° for satisfied patients. Only highly satisfied patients had an increase in ROM after TKR from 120° to 122°.

### Complications

There were eight TKR revision surgeries: two for aseptic loosening, three for prosthetic joint infection (PJI), two patellar revisions for lateral patellar instability, and one for patellar clunk syndrome. Five of the eight patients who required revision surgery managed to return to sports prior to revision.

## Discussion

The principle finding of this study was that most patients returned to sport by 6 months postoperatively (80%), with a significantly higher UCLA score than prior to surgery. Therefore, patients should expect participation in sports activities after TKR.

The most reliable predictive factors of RTS after TKR are preoperative UCLA and ASA scores and, contrary to other published studies (15, 16, 20), age, gender, and BMI appear not to influence the RTS. The absolute number of sports practiced by patients pre- and postoperatively was similar; however, there was a change in types of physical activities. This study was not the first to evaluate the RTS after TKR, but it is one of the largest and with long-term follow-up. Ten studies were used to compare to our series (Table 3). These studies had a mean follow-up >1 year, more than 100 patients (except [13]), and meta-analysis. Bradbury et al. [14] reported that 77% of patients who participated in sport preoperatively returned to sport postoperatively with or without adaptation of activities. More recently, Chatterji et al. [15] reported that 75% of patients returned to sports activities after 1 year.

**Table 3:** Return to sport after TKR, data extracted from studies included in the review (10 studies).

Study	Study design	Mean follow-up	Study population	RTS	Time to RTS	Mean post-operative UCLA	UCLA progression	Satisfied and very satisfied
Bauman et al. [16]	Cross-sectional survey	36.6 month	184 patients	–	–	6	–	–
Bonnin et al. [24]	Cross-sectional survey	44 month	347 patients	56% group <75 yo	–	–	–	83%
Bradbury et al. [14]	Cross-sectional survey	5 years	160 patients	77%	–	–	–	–
Chang et al. [10]	Retrospective study	2 years	369 patients	76%	–	4.8	+0.3	–
Chatterji et al. [15]	Cross-sectional survey	Between 1 and 2 years	144 patients	75%	–	–	–	–
Dahm et al. [3]	Cross-sectional survey	5.7 years	1206 patients	–	–	7.1	–	91%
Hopper and Leach [13]	Cross-sectional survey	21.6 months	76 patients	63%	–	–	–	80.3%
Noble et al. [8]	Cross-sectional survey	>1 year	253 patients	–	–	–	–	75%
Witjes et al. [18]	Systematic review	Variable	18 studies	36–89%	12 weeks	4.6–5.9	–	–
Wylde et al. [17]	Cross-sectional survey	>3 years	866 patients	73%	–	–	–	–

Mean postoperative UCLA score was comparable to the study by Bauman et al. [16] who found a mean score of 6.0 in a series of 184 TKR. Dahm et al. [3] reported a mean score of 7.1 with 74% of patients engaged in activities at a mean of 5.7 years after arthroplasty, with 16% of the patients reporting participating in heavy manual labor or sports deemed “not recommended” by the Knee Society survey [6].

Chatterji et al. [15] and Wylde et al. [17] have found, patients who underwent TKR reduced the intensity of high-impact activities such as jogging, skiing, or tennis, while increasing low-impact activities such as walking, hiking, cycling, or swimming. In a systematic review, Witjes et al. [18] reported that RTS for TKR varied from 36% to 89%, with mean total numbers of postoperative sports of 0.2–1.0 sports with a mean of 13 weeks for RTS.

There is no evidence of any correlation between high-level sports and early implant loosening, bearing surface wear or premature revision. Although some surgeons discourage high-impact sport, in contrast, Mont et al. [19] showed that high-level tennis players returned to tennis after TKR. Healy et al. [6] reported limited evidence-based information on implant survival after sport to make recommendations where patients are recommended to avoid high-impact sports. In our study 147 patients (33%) participated in high-impact sport without implant failure. Therefore, if patients understand the risks associated with their activities and choose to return to sport, surgeon should not discourage their patients. Kersten et al. [20] showed that almost half of patients who underwent TKR did not meet health-enhancing physical activities guidelines. In a study by Walker et al. [21] of patients with lateral unicompartmental knee replacements (UKR), the majority of patients decreased their activities to protect their UKR. In our series of patients with TKR, activity restriction because of the TKR occurred in only 22 patients for reasons of pain, knee instability, or limited range of motion.

Hopper and Leach [13] compared UKR to TKR, where UKR had superior results and they found that patients with TKR returned to low-impact sport. In the TKR group, 63.4% returned to sport by 4.1 months with a significant reduction in playing bowls and golf postoperatively. In active golfers, Mallon and Callaghan [22] found that the players’ handicap increased significantly and their driving distance was substantially reduced. Jones et al. [23] did not show an increase in revision rates due to high-impact activities at midterm follow-up. Bonnin et al. [24] have shown how satisfaction depends upon preoperative patient expectations. In our study, we have a high percentage of patients that are satisfied or very satisfied (92%) with a mean Forgotten Joint Score of 82/100. This demonstrates the improvement in quality of life as a result of TKR, which is confirmed by other studies [25].

This study has several limitations. Firstly, this work is a retrospective study, with a low level of evidence. Secondly, 7% of patients were lost to follow-up, and we could not assess if these patients were still alive or if they underwent revision surgery. In a similar study, Chang et al. [10] had a response rate of just 65%. Thirdly, the mean follow-up in this study was 43 months, which is adequate to determine early satisfaction outcomes, activity levels and time to RTS, but not long enough to evaluate revision rates, wear, and loosening.

Total knee arthroplasty is an effective treatment for pain relief; functional improvement and RTS is likely after TKR with a high satisfaction score. Preoperative condition and activity are the two most significant predictive factors for RTS. After TKR, patients should be encouraged to maintain physical activities with individual adaptions based upon general health, preoperative activity level, and type of preoperative sport. Then, surgeons should explain to the patient what kind of activities they can participate in after TKR and how to adjust their activities and give more details on expected results according to patients’ preoperative status and health.
